# Participant perspectives on incentives for TB preventative therapy adherence and reduced alcohol use: A qualitative study

**DOI:** 10.1371/journal.pgph.0002472

**Published:** 2024-04-24

**Authors:** Ayesha Appa, Amanda P. Miller, Robin Fatch, Allen Kekibiina, Brian Beesiga, Julian Adong, Nneka Emenyonu, Kara Marson, Monica Getahun, Moses Kamya, Winnie Muyindike, Michael McDonell, Harsha Thirumurthy, Judith A. Hahn, Gabriel Chamie, Carol S. Camlin

**Affiliations:** 1 Department of Medicine, University of California San Francisco, San Francisco, California, United States of America; 2 School of Public Health, San Diego State University, San Diego, California, United States of America; 3 Global Health Collaborative, Mbarara University of Science and Technology, Mbarara, Uganda; 4 Infectious Diseases Research Collaboration (IDRC), Kampala, Uganda; 5 Institute for Global Health Sciences, University of California San Francisco, San Francisco, California, United States of America; 6 Department of Psychiatry and Behavioral Sciences, Washington State University, Spokane, Washington, United States of America; 7 Department of Medical Ethics and Health Policy, Perelman School of Medicine, University of Pennsylvania, Philadelphia, Pennsylvania, United States of America; 8 Department of Obstetrics, Gynecology & Reproductive Sciences, University of California San Francisco, San Francisco, California, United States of America; South African Medical Research Council, SOUTH AFRICA

## Abstract

Economic incentives to promote health behavior change are highly efficacious for substance use disorders as well as increased medication adherence. Knowledge about participants’ experiences with and perceptions of incentives is needed to understand their mechanisms of action and optimize future incentive-based interventions. The Drinkers’ Intervention to Prevent Tuberculosis (DIPT) trial enrolled people with HIV (PWH) in Uganda with latent tuberculosis and unhealthy alcohol use in a 2x2 factorial trial that incentivized recent alcohol abstinence and isoniazid (INH) adherence on monthly urine testing while on INH preventive therapy. We interviewed 32 DIPT study participants across trial arms to explore their perspectives on this intervention. Participants described 1) satisfaction with incentives of sufficient size that allowed them to purchase items that improved their quality of life, 2) multiple ways in which incentives were motivating, from gamification of “winning” through support of pre-existing desire to improve health to suggesting variable effects of extrinsic and intrinsic motivation, and 3) finding value in learning results of increased clinical monitoring. To build effective incentive programs to support both reduced substance use and increased antimicrobial adherence, we recommend carefully selecting incentive magnitude as well as harnessing both intrinsic motivation to improve health and extrinsic reward of target behavior. In addition to these participant-described strengths, incorporating results of clinical monitoring related to the incentive program that provide participants more information about their health may also contribute to health-related empowerment.

## Introduction

Worldwide, people living with HIV (PWH) who drink alcohol heavily are at exceptionally high risk of morbidity and mortality due to active tuberculosis (TB), with over one-third of AIDS-related deaths attributable to TB and at least 10% of active TB globally attributed to alcohol use [1–3]. Unfortunately, TB preventive therapy (TPT) and concurrent alcohol use among PWH can be complicated by considerations such as hepatotoxicity and medication non-adherence [4], and both are particularly challenging to monitor in low-resource settings. Sub-Saharan Africa, and specifically Uganda, is home to some of the highest rates of alcohol consumption in the world, which has been described as “adding fuel to the fire” of the HIV epidemic in this region [5, 6]. To address these challenges simultaneously, the Drinkers’ Intervention to Prevent Tuberculosis (DIPT) trial in Uganda [7] employed a behavioral strategy that leveraged incentives to support both isoniazid (INH) preventive therapy adherence and alcohol use reduction among PWH with heavy alcohol use and latent TB infection.

Based on the principle of positive reinforcement, reinforcers (a.k.a. incentives) have been used to improve health behavior. Contingency management (CM) is one such incentive intervention originally designed to treat addiction. In CM, patients who demonstrate drug or alcohol abstinence, usually via point-of-care urine tests, receive incentives. The monitoring schedule is typically designed to maximize the chances that individuals will achieve abstinence, though authors acknowledge that abstinence is just one of many meaningful goals related to reduced alcohol use [8]. That said, CM is the most effective psychosocial intervention for establishing abstinence, including specific evidence in alcohol use disorder conducted by our group (MGM) [9–11]. Both CM and other incentive interventions with less intensive monitoring schedules have been found effective to improve medication adherence, but data are mixed and the use of financial incentives to support multiple behaviors, such as reduced substance use and increased medication adherence, has been less commonly studied [12]. The DIPT trial incentive intervention harnessed the specificity of CM in tying incentive receipt to objective measures of recent alcohol use and INH adherence and used an escalating incentive structure of CM with ongoing demonstration of target behaviors. However, because of feasibility challenges to participants in urban and rural Uganda posed by the frequent monitoring used in CM (2–3 times weekly), the DIPT intervention used monthly monitoring instead. As such, we will hereafter refer to the DIPT intervention as an incentive-based intervention, though literature pertaining to use of incentives in healthcare as well as CM specifically will be discussed as relevant.

To build effective incentive programs that support positive changes in multiple behaviors, a well-founded, participant-centered understanding of the relationship between incentives and behavior change is essential, particularly in the context of supporting vulnerable populations, such as PWH with substance use disorders in need of treatment or preventive therapy. Qualitative research of participant perspectives related to incentive receipt in programs to improve health has been limited [13, 14], particularly in low-middle income countries [15]. In this analysis we explore DIPT trial participant perspectives on the use of incentives as part of multi-target contingency management for TPT adherence and alcohol use reduction.

## Materials & methods

### Study design & setting

The parent trial for this study is the DIPT trial, a randomized, 2x2 factorial trial designed to determine effectiveness of financial incentives contingent on INH adherence and/or alcohol use for TB preventive therapy among PWH with heavy alcohol use in 4 communities in Uganda (2 urban, 2 rural). Informed by formative qualitative work exploring relationships between HIV, HIV care, and alcohol use, our study protocol and detailed methods are described in a prior publication [6, 7, 16]. Briefly, DIPT inclusion criteria comprised of the following: 1) adult living with HIV on anti-retroviral therapy (ART) for ≥6 months, 2) alcohol consumption with an AUDIT-C score 3 or above if female or 4 or above if male, indicating unhealthy alcohol (a term which encompasses any alcohol use related to health consequences including alcohol use disorder) [17], 3) having a urine ethyl glucuronide (EtG) dipstick test >300ng/mL, indicating recent alcohol use, and 4) latent TB infection with a positive tuberculin skin test and no history of prior active TB or treatment for TB. We excluded participants if they: were not fluent in either Runyankole or English, resided farther than 60 km from a study site, were currently pregnant, were on nevirapine, or had initiated dolutegravir in prior 3 months.

We randomized eligible participants to one of the following study arms: no incentives (Arm 1), incentives contingent on recent alcohol abstinence (Arm 2), incentives contingent on recent INH adherence (Arm 3), or incentives contingent on both behaviors (Arm 4). In Arm 4, behaviors were independently rewarded (e.g., a positive urine EtG did not preclude receipt of incentive earning for INH adherence). All participants were initiated on a 6-month course of INH and pyridoxine for TPT and received brief advice on alcohol use and adherence. We screened participants at week 2 and 4 and then monthly thereafter (through month six) for INH toxicities. In addition, participants in arms B-D above provided urine samples for EtG and/or IsoScreen testing at these visits, which determined incentive provision for no alcohol use and INH adherence respectively. Incentives were lottery scratch cards that corresponded to a cash prize, with values chosen in consultation with a community advisory board comprised of clinic staff, community leaders, and patients at each site. Low-value prizes were 5,000 Ugandan shillings) ($1.40 USD), high-value prizes were 50,0000 ($14.00 USD) no more than ten times this amount, and medium-value prizes were 10,000 ($2.80 USD); slightly more than half of one week’s worth of typical wages in rural Uganda [7]. Consistent demonstration of the target behavior between visits led to increasing opportunities to earn cash (by earning an increasing number of lottery scratch cards). All participants also received reimbursements for their travel to the clinic, that ranged from 10,000 to 40,000 (~$2.80–11.25 USD) Ugandan shillings depending on how far they lived from their study site.

### Qualitative sampling & recruitment

At 6 months following enrollment, participants were purposively sampled for participation in qualitative in-depth interviews with interviews collected between 7/15/2020–1/29/2021. Purposive sampling was performed to ensure equitable qualitative study of participants by strata including study arm, study site, sex (with target 25% women, reflective of PWH with latent TB and unhealthy alcohol use), and alcohol (EtG) and INH-related (IsoScreen) urine testing results, and to maximize heterogeneity in the perspectives and experiences of trial participants. In-depth semi-structured interviews sought to explore participant experiences related to the intervention and understand the role of financial incentives on heavy drinking and INH adherence, in addition to the impact of COVID-19 on economic wellbeing, HIV ART adherence, and interpersonal relationships.

### Data collection & analysis

Interviews were conducted by Ugandan research assistants trained in qualitative methods and fluent in the local language, Runyankole. With participant consent, the interviews were audio-recorded and most lasted approximately 1–2 hours (range: 44 minutes– 2 hours, 16 minutes). Audio records were transcribed and translated into English, then deidentified so participants could not be identified by transcripts. A coding framework was developed iteratively, employing deductive and inductive approaches: the first framework was created in accordance with the study research questions, then was refined collaboratively by the research team throughout the coding process. A team of researchers reviewed and coded the transcripts using Dedoose qualitative software. Final data analysis was guided by principles of reflexive, thematic analysis, described by Braun and Clark [18], with use of memo-ing to organize findings derived from deductive and inductive coding of data into nascent themes. Ultimately, data synthesis was performed with input and analytic cross-checking from both the Uganda-based and US-based research team, familiar with both participant communities and study intervention. Accompanied by supportive evidence in the form of transcript excerpts, the emergent themes are presented below. Excerpts are contextually grounded by classifying individuals’ sex, study arm, and categorical results of CM by study arm. For this analysis, we described urine test results as follows: if a participant had 0 urine samples positive for EtG or negative for INH, their alcohol use reduction and/or INH adherence was described as “excellent.” If 1–2 urine samples were EtG positive or INH negative, the assigned descriptor was “good”; if 3–5 urine samples were EtG positive or INH negative “poor”.

### Ethics approval and consent to participate

This study was approved by the Institutional Review Board at University of California, San Francisco; the Mbarara University of Science and Technology Research Ethics Committee; the Makerere University School of Medicine Research Ethics Committee; and the Ugandan National Council for Science and Technology. Informed consent was obtained from participants in their preferred language in writing prior to enrollment.

## Results

### Participant characteristics

Of 32 DIPT participants with HIV, heavy alcohol use and latent TB, 63% of participants were male (n = 20) with a median age of 38 years (IQR 31–44). We recruited half of the participants from urban and rural HIV clinics in Uganda (50% each). The number of participants recruited from each of the four study arms ranged from 7 to 9.

### Range of Initial reactions to CM

Participants met the offer of conditional incentives with skepticism and/or appreciation. Participants had a range of initial responses to learning about the trial study design and the receipt of incentives for behavior change that they perceived to be consistent with their health-related goals (either INH adherence and/or alcohol reduction). Some reported suspicion that there may be ulterior motives responsible for provision of incentives in addition to free medication (INH), with worry about being a test subject or about coercion. However, many also simultaneously or separately described experiencing a sense of warmth coming from the study health worker due to the use of incentives, as if the incentives communicated caring.

"I thought I was going to be forced to stop taking alcohol. Also, you think the people care about my health."

- Male, INH & alcohol incentives arm

(Excellent INH adherence, good alcohol use reduction)

“I wondered what they would gain and thought they wanted to just entice people to take medication. They were going to always give transport and incentives which seemed too good to be true but I decided to still join and find out […] I saw others being given cards to scratch and given money, so I knew it was not a hoax. I was very motivated to go back home and take my medication.”

- Female, INH & alcohol incentives arm

(Excellent INH adherence & excellent alcohol use reduction)

“I thought that health workers care about our lives—someone giving you a medication for free and also incentives for taking it well. I thought of stopping to drink alcohol for good, but failed."

- Female, alcohol incentives arm

(Poor alcohol use reduction)

### Expeditious spending of incentives for tangible materials

When earned, the cash prize incentives were promptly utilized with a tangible impact on participants’ quality of life. Most participants receiving incentives enthusiastically described items that they were able to purchase solely due to study participation, such as food (meat, salt, etc.), farming equipment, or personal items. Female participants were more likely to describe use of incentives to provide care or support for dependents. Participants commonly reported spending the cash incentive soon after receipt, often on the same day or on the way home following a study visit.

“These incentives have helped me a lot. I would win then I go and shop what I need at home, and on top of that they would also refund my transport to back home. I did not have a hen at home and one time I won and bought one. It became a productive one; at first it hatched 10 chicks on top of giving me eggs, and it is laying eggs again. Recently my child felt sick and I sold one chick, got money, and paid a bill for my child’s sickness. The other day I lacked salt and sold five eggs […] and I thank the entire study team.”

- Female, INH incentives arm

(Excellent INH adherence)

“On the gift they gave me, I did not have a blanket, hoe and a panga [machete] and I bought them since I had returned from the shores without anything. When my brother met me from farming, I told him that they gave me pills to prevent chest pains [INH] and incentives so I did not know how to thank them. The next time I was called upon to participate I would go running.”

- Male, INH incentives arm

(Excellent INH adherence)

### Mechanisms to enhance motivation

Some participants describe a strong sense of motivation related to winning incentives. For many participants, the opportunity to earn incentives was a clear encouraging factor in changing behavior and for some, the clear dominant motivating factor that participants described (whether or not it led to consistent behavior change). This was often described as a desire “to win”, in line with the behavioral economic concept of gamification [19]. At times, when participants expressed frustration that their motivation to win did not always work, it was noted that this was related to longer times between study visits.

“Participant (hereafter, P): I would promise myself not to drink alcohol again so as to win during the next visit, but eventually I find myself drinking alcohol again.

Interviewer (hereafter, I) I: Okay, please tell me when you were first approached by the study team member and told that you would be offered a reward for reducing the amount of alcohol you take, what were your first thoughts?

P: I thought of leaving alcohol for good to be able to win an incentive, but I failed.”

- Female, alcohol incentives arm

(Poor alcohol use reduction)

"I: What was your main reason for stopping to drinking alcohol?

P: The incentives… it greatly influenced me."

- Female, INH & alcohol incentives arm (excellent INH adherence, alcohol use reduction)

Other participants describe incentives as important for preserving their health. Certain participants characterize their interest in behavior change as a combination of both the desire to win and/or earn money in addition to the positive health-related change they wanted to make for themselves.

“P: I said to myself that this one is aiming at making [me] stop to drink alcohol. I felt happy about those incentives and promised myself to reduce on the alcohol I take to be able to win. The first time visit I did not win, but the second time I won… 30,000 shillings. The next time I forgot, and towards the clinic visit I took some alcohol and I did not win, and I felt bad about it.

I: For that one time when you won, what motivate you to reduce alcohol?

P: I wanted to win, and also know that alcohol is harmful to my health.”

- Male, alcohol incentives arm

(Very poor alcohol reduction)

“I: How did you feel about the reward received for your negative test results?

P: I felt happy about it and thought that if I stop drinking alcohol for good then even incentives will increase. And I really did it, and that is what happened.

I: What are the main reasons you were motivated to reduce on the alcohol?

P: I knew I would get many incentives and I would also get better health-wise.”

- Male, alcohol incentives arm

(Good alcohol reduction)

Other participants prioritized intrinsic motivation of health and de-emphasized the role of incentives. Some felt that earning incentives via CM was not a meaningful contributor to INH adherence and/or alcohol use reduction, with health as a more important motivating factor than earning incentives.

“I: You mentioned earlier reducing your alcohol intake being motivated by INH therapy. Is there any other reason?

P: No, I had lost hope because of stress.

I: How did it influence your decision to take alcohol?

P: It did influence me, but it was more so because of my great fear of TB. The incentives were okay, but also we were counselled and helped, so I feel fine, I sleep better now.”

- Female, alcohol incentives arm

(Excellent alcohol use reduction)

“I: After getting the incentive during your first clinic visit, what did you think at the time that would happen when you took the test at the next study visit?

P: When the time would clock, I would think about getting the medication as the basic thing […] What motivated me to take well the pills were not the incentives but to have good health. I would still take the medication even if there were no incentives. But I would feel happy after getting the incentives for INH adherence.”

- Male, INH incentives arm

(Excellent INH adherence)

Finally, others described the interconnected nature of the study experience, including their relationships with health workers, the incentives, and the health-related changes they made as all part of their ongoing motivation or engagement. For many participants, factors associated with behavior change and the motivation to change were not clearly defined or partitioned, but rather multiple aforementioned factors comingled.

“I: So you were motivated to take INH. Was there anything else that motivated?

P: The Incentive, then medication to prevent TB but also the study staff was good in counseling. Even when you come late, they engage us and are calm.”

- Female, INH & alcohol incentives arm

(Excellent INH adherence & excellent alcohol use reduction)

"P: I did not know that alcohol is that harmful to one’s health […] I reduced on the alcohol I take after joining this study and when the health worker of this study told me that alcohol may damage my liver. Also whenever I would come for clinic visit, and they test me and found out that I have been taking alcohol, I would not win any money and I never wanted to miss getting that money. During the time I have spent on this medication, to tell the truth I reduced taking alcohol."

- Female, INH & alcohol incentives arm

(Excellent INH adherence & good alcohol use reduction)

Many reported that INH adherence was not difficult, as they were used to taking medications for HIV or simply didn’t feel the need to comment. Others describe challenges related to remembering to take more than one medication daily, privacy when needed to take it in a work context, or adverse effects (including descriptions of peripheral neuropathy and dyspepsia related to concurrent alcohol use). Additionally, a few participants explained that there are challenges related to INH adherence: because it is a medication for infection prevention, patients cannot feel the clinical improvement they gain from TB preventive therapy, in contrast to ART for HIV/AIDS.

“You know when you are not sick and someone tells you that he has treated you, it is as if they are lying you, you see… Like how I told you earlier, when get treatment when you are not sick, you may not know its value, and sometimes I would wonder, why I should come and get this medication and miss going to work because I was not sick… And the fact that the medicine was for prevention, I would say okay let me get it and take it so that I don’t regret in future after developing tuberculosis."

- Female, standard of care arm

Almost all participants described social pressure as a barrier to reducing alcohol use or as a trigger for returning to using alcohol. Many participants also discussed the element of financial compulsion, with difficulty when others purchased alcohol for them. This also added pressure for women, for whom it was common and socially acceptable to drink alcohol that men bought for them.

“[…] Then he buys me waragi [a local alcohol-containing drink] and we drink, and I tell him that alcohol has failed me to get incentives at the clinic where I get my medication […] [I] tell him that whenever I reach at the clinic, the health workers talk badly after discovering that I have continued to drink a lot of alcohol.”

- Female, alcohol incentives arm

(Poor alcohol use reduction)

“I would sometimes sit and tell God, that you see alcohol is turning me into another thing, please help me and stop drinking alcohol. But because of peer influence, my friends could not allow me [to] stop. They would find me there and tell me, ‘let’s go and I buy for you alcohol’, and I find myself resuming to drink again—you know if you are not the one to pay the bill you can drink as much as you can. But ever since I started on this medication, I have reduced on the alcohol that I take.”

- Male, alcohol incentives arm

(Poor alcohol use reduction)

That said, most study participants reported reducing alcohol use, and multiple participants described completely abstaining from alcohol following study participation.

“I used to drink before joining this study, you know when you an alcohol drinker you cannot even think of saving, I would go work like weeding in someone’s plantation and earn 3,000 [Ugandan shillings: ~US$0.85 in 2020] per day worked, then I pass by a bar and consume them all when I am not even having salt at home. So, when I joined this study, it helped me a lot […] I stopped drinking alcohol and now I spend the money that I used to drink to buy household needs and this has made me realize that alcohol is a bad thing.”

- Female, INH incentive arm

(Excellent INH adherence)

Separate from incentives, participants reported that one powerful aspect of study involvement was the positive feedback they received from having normal liver enzyme tests: they liked receiving personalized information about their bodies (e.g., urine testing, organ function) and providing a specific description of how it led to “courage” related to health. One participant noted the relationship between lab testing, health and wanting to preserve health:

"And I had also developed the courage about my health after testing and being told that my organs function well, because when you are tested and they tell you that you see your body systems are not functioning well, you may resort to drinking alcohol after all you would be having nothing to protect." Male, alcohol incentives arm (Excellent alcohol use reduction).

A woman noted that after receiving results that her liver/kidney was normal, “I would make me feel less worried about my health and it has given me courage to continue taking the medication [INH].” INH incentives arm (Excellent INH adherence).

## Discussion

We found multiple actionable themes that may be helpful to leverage in future incentive-based programs, primarily related to 1) incentive amount and spending, 2) self-report of mechanisms mediating incentive receipt and behavior change, and 3) specific findings related to preventative medication and/or alcohol-related behavior change. Participants almost universally reported expeditious spending of their earned incentives, which were sized to purchase items that had the potential to improve quality of life, which likely reinforced the target behavior (short-term INH adherence and/or alcohol abstaining). Participants strongly associated their behavior with enhanced agency to purchase tangible items, including food, farming equipment, items for their children, etc., that they described as bringing happiness and practical support to their lives. This is consistent with previously reported participant perspective on financial incentives for HIV testing in Kenya: receiving incentives may have empowered participants to prioritize health, as the incentive for health improvement was aligned with subsistence and practical needs [20]. Moreover, as expenditures were also somewhat gendered, with men more focused on purchase of tools and equipment, and women on food and items for children, incentives offered an opportunity for social role fulfillment via provision of these items for their households. Additionally, participant-reported effectiveness associated with a sufficiently-sized incentive is consistent with a considerable body of prior CM literature in which increasing incentive size is associated with a dose-response effect on CM effectiveness, regardless of the substance studied or behavior incentivized [21–23]. Finally, while immediacy of incentive provision is considered key to CM efficacy [21, 24], prompt spending of the incentive has been associated with more pronounced behavior change (compared to saving) [25]. Participant report of great satisfaction with spending their incentive on key items immediately, often on the way home from their study visits, is consistent with those prior findings, and highlights the importance of careful selection of incentive type and size in future incentive-based interventions.

Regarding participant-perceived mechanism of behavior change related to incentives, there was a spectrum of expression around motivating factors. Some participants reported a clear desire to drink less and/or to adhere to INH in order “to win”–expressly to achieve or earn incentives. Other participants described that earning incentives was a positive factor in leading to behavior change, but that they also felt motivated by changing their health and their intrinsic desire to make change on health-related goals concordant with the study. Yet another group of participants de-emphasized the importance of incentives, feeling that their health was the primary motivator. A final category of participants mentioned a combination of factors difficult to separate from one another, including both intrinsic motivation, incentive-related motivation, as well as other factors like relationships with study health workers, etc.

Gamification, a concept utilized in the behavioral economic field with application across disciplines, refers to the application of common elements of game playing to drive engagement [19, 26]. While CM primarily harnesses principles of operant conditioning (i.e. rewarding rather than punishing) to drive behavior change, for those participants that clearly enjoyed or identified with the concept of “winning,” aspects of CM that may overlap with gamification, such as use of a variable incentive (in this case, lottery scratch cards) and increasing ability to earn incentives conditional on meeting CM goals (that may correspond with “levels” of a game) may be particularly effective.

The participants who referenced intrinsic motivation in relation to incentive provision touched upon a topic that has long been one of consideration in the CM literature. Behaviorists have described mixed evidence about whether incentive provision as an external reward may negatively impact intrinsic motivation to change [27], though a critical review of studies of health behavior reinforcement found that reinforcement may lead to feelings of competence, thereby increasing intrinsic motivation [28]. In our study, the most salient perspective was one of equanimity (e.g., participants noting satisfaction with the incentive alongside report of their own desire to change, without evidence that CM was affecting intrinsic motivation positively or negatively). This is consistent with prior research showing inconclusive data on the effect CM on intrinsic motivation [29]. However, consistent with prior study [14], these data support carefully selecting incentives aligned with the participants’ sociocultural context and with participants’ own health-related goals.

This study suggests that multiple overlapping spheres contribute to medication adherence and alcohol use reduction in PWH with unhealthy use, with several novel findings related to incentive receipt. Both adherence to TPT and alcohol use reduction among PWH on ART are governed by multiple biopsychosocial factors; participants shared a spectrum of influences, from self-empowerment related to prior success in the context of ART adherence to ongoing challenges related to privacy, side effects, and social pressure. However, several participants described the challenge of medication adherence for a preventative health condition, in which there is no clear physiologic improvement from preventive therapy that the participant perceives. In other words, use of an incentive-based intervention may be particularly helpful when there are not positive physical improvements expected to serve as positive feedback to the participant. This suggests a potential mechanism for effectiveness of incentive-based interventions for medication adherence in TPT, a strategy previously found to be efficacious in a small number of trials [30–32], and supports its ongoing study and dissemination to other preventative interventions. Interestingly, a few participants did ascribe physical benefit to INH adherence; it is possible that concurrent alcohol use reduction related to the study led to perceived improved health. Relatedly, multiple participants raised the issue of valuing the receipt of positive feedback on their health as part of this study, especially the liver enzyme tests performed to monitor for INH hepatotoxicity. While lab testing among PWH is likely less frequent and thus relatively noteworthy in this resource-constrained setting, many voiced a consistent message that learning about healthy liver function was extremely valuable. Knowledge of normal liver function may provide reassurance that reducing alcohol use may yet lead to benefit and is not a futile endeavor. Based on participant description, this knowledge also specifically led to feelings of health-related courage or empowerment. In a social context that involves pervasive stigma related to HIV, TB, and/or substance use, this bolstering of courage through increased transparency could translate into self-efficacy thereby influencing motivation.

Limitations of this study include its cross-sectional nature, inability to account for social desirability bias [33], and generalizability. Our data consisted of one in-depth interview, so observations are cross-sectional. However, interviews were collected at 6 months following enrollment, as participants were completing INH treatment, which was an optimal time to assess their views about progress made during the study. Another important limitation is the role of social desirability bias, as participants may have presumed that study staff would like to hear positive feedback. However, our findings that explore various relationships between incentives and motivation suggest that many participants felt comfortable attributing their success (or challenge) to factors outside the study. Finally, our objective was to explore participant perspectives of incentive receipt related to reduced substance use and/or optimal adherence, and while many of these beliefs could be externally valid in multiple social or clinical contexts, it may be difficult to generalize outside the participant experience in Uganda, including to those PWH not on ART. However, a key strength of our study is that it is one of the few qualitative explorations of participant perspectives on incentive receipt during an incentive-based intervention in a low-resource setting.

## Conclusions

In this study of participant perspectives on the use of financial incentives for short-term TB preventive therapy adherence and alcohol abstaining we conclude that: 1) carefully selected incentives with sufficient size led to participant satisfaction, and 2) multiple mechanisms appeared to promote behavior change. In addition to incentives, participants valued elements that increased transparency about their health which led to health-related empowerment.

## Supporting information

S1 Checklist Inclusivity in global research. (DOCX)
